# What is the best way to collect urine from neonates? Comparison of the success rate and volume using different methods in a repeated measures design

**DOI:** 10.1186/s13104-026-07971-8

**Published:** 2026-08-11

**Authors:** Jochen Kittel, Alexandra Peiper, Michael Melter, Susanne Brandstetter

**Affiliations:** 1 Children’s Hospital (KUNOClinics), Hospital St. Hedwig of the Order of St. John, University of Regensburg, Regensburg, Germany; 2 Department of Otolaryngology, Head and Neck Surgery, University Hospital Augsburg, Augsburg, Germany; 3 Research and Development Campus Regensburg (WECARE), Hospital St. Hedwig of the Order of St. John, Regensburg, Germany; 4 Children’s Hospital (KUNOClinics), Clinic St. Hedwig, University of Regensburg, Regensburg, Germany

**Keywords:** Neonates, Urine collection, Methods, Stimulation manoeuvre, Volume

## Abstract

**Objective:**

Urine collection from neonates can be difficult. We therefore designed a study to compare success rates and effectiveness of different urine collection methods in neonates.

**Results:**

Urine collection by compress and by sponge methods were unsuccessful and not further evaluated. Data was obtained from 23 neonates for analysis of effectiveness of the other methods of urine collection. The cotton wool pad achieved the most measurable samples of urine (82%). Wet diaper had a success rate of 78%, clean catch after bladder stimulation 70% and the adhesive urine collection bag recorded success rate of 56%. Differences observed among the success rates between the four methods were not statistically significant. There was notable variability in urine volume collected, with the clean-catch method yielding a median of 7.0 mL and the cotton wool pad only 1.0 mL.

## Introduction

Urine samples play a crucial role in clinical practice for diagnosing urinary tract infections (UTIs), metabolic disorders, glomerular diseases, evaluating tubular function, and conducting epidemiological studies on environmental chemicals. Selecting the appropriate urine volume and collection method is therefore essential. The diagnosis of UTIs, particularly as a cause of fever in neonates and infants, is often addressed in clinical settings, where the absence of leukocytes or nitrites in urine can help rule out these infections [1–3]. Rapid urine test strips can provide this sufficient diagnostic information with minimal urine volume, reducing the need for further invasive examinations [1, 3].

However, obtaining uncontaminated urine samples from neonates and non-toilet-trained infants is challenging, and contamination is a common issue. Preferred collection methods include clean catch, urine collection bags, in-out catheterization, and suprapubic aspiration [1–3]. While established methods like suprapubic puncture have high success and low contamination rates, they are invasive and carry risks [4]. Conversely, clean catch and urine collection bags, though less invasive, have lower success rates and higher contamination risks, with contamination rates in bags being as high as 37–87% [5–11].

Various techniques have been used, including compresses, sponges, rapid urine tests, and cotton wool pads. To the best of our knowledge, these methods have not yet been compared in a single study on infants under 6 months of age together with the current frequently used urine collection bags [2, 12, 13]. Therefore, this study aims to test and compare six different urine collection methods specifically for neonates.

### Methods

This study employed a prospective within-subject repeated-measurement design to evaluate six urine collection methods. The tested methods differ in their difficulty, but all of the proposed methods can be learned with a qualified instruction in a short period of time [2]. All neonates hospitalised in an intermediate care unit for conditions like low birth weight or jaundice were eligible for participation; excluding those with UTIs, known urinary tract anomalies or high-flow therapy.

Informed consent to participate was obtained from the parents or legal guardians of all participants. The study was approved by the Ethics Committee of the University of Regensburg.

Due to the high staffing requirements and the presumed lack of parental acceptance of the study, we aimed for a sample size of 50 participants. These concerns were confirmed, so data collection continued from February 2017 to March 2018. We used six different techniques for urine sampling (described in detail in Table 1): an adhesive urine collecting bag, a compress in the diaper, a sponge in the diaper, a quick urine test (Combur-Test^®^) pressed in the wet diaper, a clean-catch after bladder stimulation and a cotton wool pad. We applied the urine collection methods sequentially to the same neonate. First, we attempted to collect urine within a urine collection bag (a standard procedure was written down and trained). This method was repeated up to three times in the event of failure, as this is the standard procedure for urine collection at our clinic. At the start of the study, it was the method with the highest level of acceptance among the nursing staff. In order to compare the success rates of the different methods, only the first attempt with urine collection bag was considered. Successful urine collection was defined as obtaining a sufficient amount of urine for laboratory diagnosis, equivalent to one millilitre. Then, the other methods were applied just once, one after the other as it was possible during the patient´s usually short hospital stay. There was no fixed order, and the aim was to test all six methods on every neonate in the shortest possible period of time. This process depended on staffing availability and the children’s clinical condition. A break of at least 3–6 h was scheduled between the individual tests. For example, newborns were not specifically woken up to perform the urine collection procedure; instead, as part of routine clinical care, they were only diaper changed, and a urine sample was collected from the diaper/ pad.

**Table 1:** Six techniques for urine sampling

Method	Description/materials	Possible contamination
Urine collection bag	Bag with glue (attached to the genital, over the external urethral orifice)	Possible contamination due to bacteria; possible pollution due to glue/ plastic bag
Compress in the diaper	A compress (“Gazin Mullkompresse”) is placed in the clean diaper, just in front of the external urethral orifice	Possible contamination due to bacteria; possible contamination with stool; possible pollution due to compress material
Diaper	The urine quick test (Combur-Test^®^) is pressed firmly onto the wet spot of micturition in the diaper at the unit	Possible contamination due to bacteria; possible contamination with stool; possible pollution due to diaper material
Sponge in the diaper	Piece of sterile sponge, taken from a vacuum dressing, is placed in the diaper, just in front of the external urethral orifice	Possible contamination due to bacteria; possible contamination with stool; possible pollution due to sponge material
Clean-catch after bladder stimulation	30 min after cessation of food intake, the neonate is held up under the arms by one person (for example caregiver), meanwhile another person carries out the stimulation manoeuvre, which consists of alternating light tapping on the suprapubic region of the abdomen and circular massage of the lumbar back section; stimulation manoeuvre is repeated in sections of 30 s until micturition occurs or the manoeuvre is stopped after a maximum of five minutes	Rarely, but possible contamination due to bacteria, especially in uncircumcised boys
Cotton wool (sanitary) pad	The cotton wool (sanitary) collection pad is stuck directly into the diaper; first check after 10 min, then after 20 min and finally 30 min after initial sticking	Possible contamination due to bacteria; possible contamination with Stool; possible pollution due to cotton wool

During the study it became apparent early on that the compress (28 tries, 4 successful) and sponge (22 tries, 0 successful) had such low success rates, that they were no longer used after subject 32. Only subjects who had measurable results from the remaining four main methods (i.e. a urine collection bag, a cotton wool pad, quick urine test (Combur-Test^®^) pressed in the wet diaper and a clean-catch after bladder stimulation) were included in the statistical evaluation. This resulted in 23 test subjects for further statistical analysis regarding the success rate and urine volume obtained.

Success rate was defined as the rate of subjects with an amount of urine sufficient for conducting the quick urine test (Combur-Test^®^) and volume of urine obtained was measured in a urine monovette in mL.

Data were collected pseudonymously and analysed using SPSS v25. Study demographics were reported as medians and interquartile ranges. Cochran’s Q test was used to compare success rates across methods. Differences between male and female children were analysed in post-hoc tests stratified for sex. Urine volumes from varying methods were compared using repeated measures ANOVA.

## Results

Fifty neonates participated in the study, which involved 209 trials for urine collection. Complete data on four collection methods was available for 23 neonates (study population, Table 2) with 92 trials. The cotton wool pad achieved the highest success rate in the series of tests with 82% successful attempts (see Table 3). The lowest success rate, apart from the two excluded methods, was achieved with the urine collection bag at 56%. There was no statistically significant difference in the success rate among the four methods (*p* = 0.20).

**Table 2:** Study population

	*N* = 50 Study population with data from any urine sampling method	*N* = 23 Study population with data from 4 different urine sampling methods
Age at investigation, median (IQR)	2 weeks (1–7)	3 weeks (1–7)
Gestational age in weeks, median (IQR)	34 weeks (28.5–35.1)	32 weeks (28-34.5)
Weight in grams, median (IQR)	2525 g (2306–3050)	2740 g (2450–3025)
Birth weight in grams, median (IQR)	1920 g (1310–2530)	1780 g (1120–2490)
Gender: male/ female (%)	30/20 (66% vs. 34%)	14/9 (64% vs. 36%)

*IQR* Interquartile range

**Table 3:** Overall comparison of the four different methods, 23 subjects were included with complete data at urine collection for all four main methods

	Urine collection bag	Cotton wool (sanitary) pad	Diaper	Clean-catch after bladder stimulation
Number of attempts (*N*)	23	23	23	23
Successful attempts (N)	13	19	18	16
Success rate (%)	56	82	78	70
Success rate female patients (%)	44	89	55	78
Success rate male patients (%)	64	79	92	64
Volume of urine (mL) (when successful) M, SD /MD (IQR)	5.2 (4.1)/ 4.0 (1.2-9.0)	1.4 (0.9)/ 1.0 (0.8-2.0)	/	6.5 (3.2)/ 7.0 (5.0-8.4)

*IQR* Interquartile range, *SD* Standard deviation *M* Mean, *MD* Median

However, sex-dependent differences emerged, with males achieving a 64% success rate with the bag compared to 44% for females; conversely, females had higher success rates with the cotton wool pad (89% vs. 79%) and stimulation manoeuvre (78% vs. 64%).

In successful collections, the average urine volume was highest with the stimulation manoeuvre (6.5 mL) and lowest with the cotton wool pad (1.4 mL). A statistically significant difference in urine volumes among methods was noted (*p* = 0.039), specifically between the cotton wool pad and the stimulation manoeuvre (*p* = 0.04) in favour of the stimulation manoeuvre. However, the difference was not statistically significant when the urine stimulation manoeuvre and urine collection bag (*p* = 0.366) and the cotton wool pad and urine collection bag (*p* = 0.058) were compared.

## Discussion

Collection of urine samples from neonates can be challenging due to low success rates and insufficient volume obtained. Our study tested six different collection methods. Sponges and compresses were deemed unsuitable due to very low success rates. This could be due to the compresses drying out too quickly or sponges absorbing too much urine. This is particularly relevant because, physiologically, newborns like in our cohort, often produce only small volumes of urine. Among the four remaining methods, cotton wool pads had the highest success rate at 82%, while urine collection bags had the lowest at 57%, though differences were not statistically significant.

With all three methods evaluated for volume (cotton wool pads, urine collection bags and stimulation manoeuvres), at least a minor amount of urine could be obtained when successful. The maximum average amount of urine was obtained via the stimulation manoeuvre. The stimulation manoeuvre for clean catch urine collection in neonates and infants has been recently shown to be both effective and feasible in routine care following fluid intake [7, 14, 15]. Several studies suggest that this manoeuvre is at least as effective as traditional noninvasive methods; however, its practicality may vary depending on available personnel and the number of subjects involved [15–17]. While data indicate it as a suitable option for urine collection in everyday clinical practice or for urine studies, the heterogeneous nature of existing studies precludes a definitive recommendation for or against its use [1–3, 13–16].

Although the success rate with the urine collection bag was relatively low with the first attempt, a high volume of urine could also be obtained if successful. However, it is cost-intensive overall, as in addition to the material costs high personnel costs are incurred because of possible repeated tests.

The cotton wool pad has high success rates, is easy to use, is very well tolerated by parents and the medical team but only produces relatively small amounts of urine. The minimum urine volume that is regularly required for further analyses is 5 mL, which was not achieved in our study via the cotton wool pad.

The problem of a small amount of extracted volume when the cotton wool pad is used has also been addressed by other authors [13].

Methods such as gauze and cotton pads are criticised for compromising urine analysis quality, particularly in observing crystals and casts that may become trapped in their fibres. Collecting urine directly from diapers was noted as a method that minimises contact and produces some successful outcomes. However, it has significant limitations, including contamination risk and low volume, making it suitable primarily for UTI screenings [13, 14, 17]. Despite some positive results from this method, further investigation with alternative techniques is required, and our study also demonstrated a surprisingly low success rate for this approach.

## Conclusion

The compared techniques for urine collection in neonates showed differences in terms of the success rate and volume obtained. Methods that have thus far played only a minor role in clinical practice, such as the cotton wool pad or clean catch urine after stimulation manoeuvre, should be given more consideration for urine collection in neonates.

### Limitations

There are certain limitations to our study. Initially, we designed a study in which 50 subjects underwent repeated measurements. However, only 23 out of 50 initially included participants had data on four urine collection methods that could be compared. This small size hampers the ability of the study to draw generalisable conclusions about the efficacy or practicality of the different urine collection methods discussed.

Secondly, we could not evaluate whether urine samples were contaminated, therefore, it is not possible to conclude if any of the methods are adequate for culture.

## Data Availability

The data are available upon request from the authors.
